# Cu_2_O and Au/Cu_2_O Particles: Surface Properties and Applications in Glucose Sensing

**DOI:** 10.3390/s121013019

**Published:** 2012-09-26

**Authors:** Yu-Ho Won, Lia A. Stanciu

**Affiliations:** 1 School of Materials Engineering, Purdue University, West Lafayette, IN 47907, USA; E-Mail: ywon@purdue.edu; 2 Birck Nanotechnology Center, Purdue University, West Lafayette, IN 47907, USA

**Keywords:** oxide, sensor, surface chemistry

## Abstract

In this work we investigated the surface and facet-dependent catalytic properties of metal oxide particles as well as noble metal/metal oxide heterogeneous structures, with cuprous oxide (Cu_2_O) and Au/Cu_2_O being selected as model systems. As an example of application, we explored the potential of these materials in developing electrocatalytic devices. Cu_2_O particles were synthesized in various shapes, then used for testing their morphology-dependent electrochemical properties applied to the detection of glucose. While we did not attempt to obtain the best detection limit reported to date, the octahedral and hexapod Cu_2_O particles showed reasonable detection limits of 0.51 and 0.60 mM, respectively, which are physiologically relevant concentrations. However, detection limit seems to be less affected by particle shapes than sensitivity. Heterogeneous systems where Au NPs were deposited on the surface of Cu_2_O particles were also tested with similar results in terms of the effect of surface orientation.

## 1. Introduction

In this work we investigated the surface and facet-dependent catalytic properties of metal oxide particles as well as noble metal/metal oxide heterogeneous structures, with cuprous oxide (Cu_2_O) and Au/Cu_2_O being selected as model systems. As an example of application, we explored the potential of these materials in developing electrocatalytic devices.

While conventional methodologies of material synthesis and processing have been developed in the past decades to a level allowing production of an enormous range of sophisticated products, pushing the state of the art forward requires understanding of current materials from new, still unexplored, perspectives. In the recent years, many studies have been carried out on size and shape control of inorganic crystals [1–10]. Now that the capacity to control shape of various particles has been established, there is a need to extend beyond synthesis and characterization of their physical and chemical properties into more rational and systematic studies on how these advances on shape and morphology control can be harvested towards the next leap in the state of the art of their applications. Systematically correlating the surface, interfacial and facet dependent properties with the discovery of new materials and their implementation into applications, such as photocatalytic or electrocatalytic devices, holds promise for the engineering of the next generation of materials with superior, not previously attained properties. To rationally design materials with built-in photocatalytic and electrocatalytic activity tailored to address emerging environmental and biomedical challenges (e.g., how to design custom materials used to decompose organic pollutants of any chemical structure), there is a need to understand how the facet dependent properties of metal oxide particles affect their catalytic activity. While there has been much research in the area of material synthesis at different scale lengths, there is not much attention given to the investigation of facet-dependent properties of shape-controlled metal oxide crystals to date. For example, a lot of attention has been given to measurements of electrical properties of nanowires [11–15], or the catalytic properties of spherical nanoparticles [16–18], but the effect of facet surface in Cu_2_O nanocrystals and/or particles with controlled shapes (e.g., cubic, octahedron, truncated octahedron or hexapod) has not been largely or systematically investigated. Synthesis of shape controlled Cu_2_O nano- and micro-crystals has already been performed [19–28], however not much attention has been given to actually exploiting this well established capacity to control this material's shape and surface properties for any biosensing or biocatalytic applications. In this work, we performed experiments that work towards closing this knowledge gap by focusing on Cu_2_O as a model metal oxide that has promising electrical characteristics, photocatalytic properties, and that is amenable to shape-controlled synthesis, but for which very little is known about the mechanisms involved during its use in electrocatalysis. Such metal oxides are interesting substitutes for noble metal catalysts owing to their lower cost, coupled with a significant catalytic activity. Among semiconductor materials, Cu_2_O has attracted intensive attention because it can be used in various devices, such as photoelectrochemical cells, sensors, catalysts, and batteries [7–10]. The morphology of Cu_2_O can be adjusted to tailor its properties to the requirements of each application. The surfaces of Cu_2_O crystals showing different morphology are exposed with different crystallographic planes. For example, the {100} crystallographic planes are exposed in the Cu_2_O cube, while the {111} planes are exposed in the Cu_2_O octahedron. These surfaces have different atomic arrangements and different surface energies, and thus they are expected to induce different physical and chemical properties.

This work establishes an understanding of the correlation between the facet-dependent properties of shape-controlled metal oxides and their resulting catalytic properties. We focused our initial work on Cu_2_O particles as a well studied model, identified the nature of reactive species involved, and established the mechanism of action. The results also determined the way that shape, surface orientation and interfacial reactivity of Cu_2_O particles affect the catalytic properties and how these can be exploited in practical devices and applications. As an example of application that will have both relevance to human health and the capacity to highlight the surface-dependent properties of differently shaped crystals glucose detection was performed. The knowledge acquired can impact the environmental and biomedical fields, and industries that are heavily using catalyst technologies.

## 2. Experimental Section

### 2.1. Materials

To synthesize Cu_2_O and Au decorated Cu_2_O (Au/Cu_2_O) particles, CuCl_2_, NaOH, sodium dodecyl sulfate (SDS), NH_2_OH·HCl, and HAuCl_4_ were purchased from Sigma Aldrich (St. Louis, MO, USA). D-(+)-Glucose, K_4_Fe(CN)_6_, and K_3_Fe(CN)_6_ for the glucose sensor tests and KH_2_PO_4_ and K_2_HPO_4_ for the preparation of the phosphate buffer solution (PBS) were also obtained from Sigma Aldrich.

### 2.2. Synthesis of Cu_2_O and Au/Cu_2_O Nanoparticles

Cu_2_O particles with various morphologies such as cube, truncated octahedron, octahedron, and hexapod were synthesized according to the report by Ho and Huang [9]. For the preparation of cubic Cu_2_O particles, 0.1 mL of 0.1 M CuCl_2_ was injected into 9.5 mL of deionized (DI) water. One M NaOH (0.2 mL) and SDS (surfactant, 0.087 g) were added into the CuCl_2_ solution under vigorous stirring. After dissolution of SDS, 0.1 mL of 0.2 M NH_2_OH·HCl was added in the mixed solution. The solution was stirred at 800 rpm for 2 h for aging. The solution was centrifuged and washed by ethanol at 5,000 rpm for 5 min to remove unreacted chemicals. Finally, Cu_2_O particles were dispersed in ethanol. Cu_2_O particles with various shapes were obtained by control of amounts of DI water, NaOH, and NH_2_OH·HCl as shown in Table 1. Au/Cu_2_O particles were prepared by adding HAuCl_4_ solution in the Cu_2_O colloid solution [29]. A volume of 50 μL of 1.2 mM HAuCl_4_ was injected into 150 μL of Cu_2_O colloid solution dispersed in DI water under stirring at 600 rpm. After stirring for 2 min, the mixed solution was centrifuged and washed by ethanol two times. Finally, Au/Cu_2_O particles were dispersed in ethanol. In this reaction, a reductant for Au is not required because AuCl_4_^−^ ions reduced by Cu_2_O. The reaction is as follows [29]:

(1) $$3{\text{Cu}}_{2}\text{O}+{{2\text{AuCl}}_{4}}^{-}+6{\text{H}}^{+}=6{\text{Cu}}^{2+}+2\text{Au}+3{\text{H}}_{2}\text{O}+8{\text{Cl}}^{-}$$

### 2.3. Apparatus and Measurements

Scanning electron microscopy (SEM) images of Cu_2_O and Au/Cu_2_O particles were obtained using a XL 40 (FEI) instrument operating at 10 kV. The X-ray diffraction (XRD) patterns of the particles were measured with a Bruker D8 focus (Cu Kα radiation, λ = 1.5406 (X001FA)(x000B4)). The electrochemical measurements of the sensors for the detection of glucose were performed with an epsilon C3 cell stand (BASi). A conventional three-electrode system, which consists of screen printed electrode (SPE, area = 4 × 5 mm^2^, Pine Research Instrumentation, Durham, NC, USA) as a working electrode, Pt wire as a counter electrode, and Ag/AgCl electrode as a reference electrode, was used for the sensor measurements.

### 2.4. Preparation and Characterization of the Sensors

As an example of application that has the potential to highlight the surface-dependent properties of differently shaped crystals, an electrochemical biosensing configuration in which Cu_2_O is the main sensing element was tested. The as-synthesized Cu_2_O particles were coated on commercially available screen printed electrodes (SPE). Cu_2_O particles dispersed in ethanol were dropped on the surface of SPEs and dried for 1 h at 25 °C. A PBS solution (0.1 M) containing KH_2_PO_4_ and K_2_HPO_4_ was prepared. Amperometric responses of the sensors were obtained in 3 mL of PBS (0.1 M) containing 5 mM [Fe(CN)_6_]^3–^ by adding glucose solution, under magnetic stirring (150 rpm). The current response of amperometric sensors usually depends on the applied potential and pH value of buffer solution. To optimize the current response of the sensor, the effects of an applied potential and a pH of the buffer solution were investigated. To confirm reproducibility of electrodes, we prepared three of the same electrodes and measured the current response three times. The pH value of the buffer solution was adjusted from 5 to 10 by adding HCl and NaOH to the buffer solution. To obtain the detection limit, the current responses of the sensor were measured by adding 1 mM glucose successively into the same measuring cell, under optimum conditions.

## 3. Results and Discussion

It is important to note that the current work doesn't aim to obtain the best sensor performance reported, but to verify the hypothesis that the surface properties of variously shaped particles influence their catalytic efficiency in both metal oxide particles, as well as in noble metal/metal oxide heterogeneous structures, with Cu_2_O and Au/Cu_2_O being selected as model systems. As an example of application, we explored the potential of these materials in developing electrocatalytic devices. To test this hypothesis, the morphology-dependent electrocatalytic properties were evaluated for both systems.

Cu_2_O particles having various shapes were prepared in house, according to the reported method by Ho and Huang [9]. The amounts of precursor materials such as NaOH and NH_2_OH·HCl were modified from the reported method to obtain well defined shapes as shown in Table 1.

**Figure 1.**
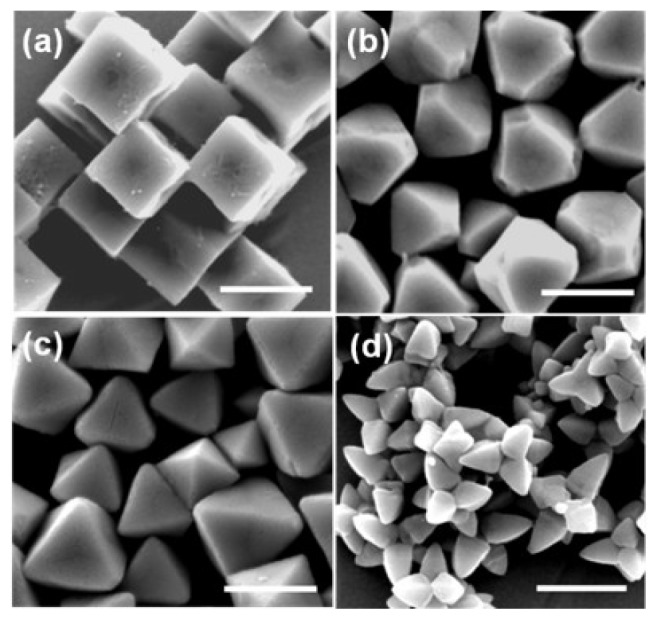
SEM images of synthesized Cu_2_O particles (**a**) Cubes; (**b**) Truncated octahedral; (**c**) Octahedra; and **(d)** Hexapods (Scale bar: 1 μm).

Figure 1 shows SEM images of synthesized cubic, truncated octahedral, octahedral, and hexapod Cu_2_O particles. The sizes of the particles regardless of shapes are in the range of 800 nm–1 μm. Cubic Cu_2_O particles Figure 1(a) have all six {100} faces and truncated octahedral Cu_2_O particles Figure 1(b) have six {100} faces and eight {111} faces. Octahderal Figure 1(c) and hexapod Figure 1(d) Cu_2_O particles have eight and twenty four {111} faces, respectively. Thus, the effects of crystal facets of Cu_2_O particles on biosensing properties can be tested.

**Figure 2.**
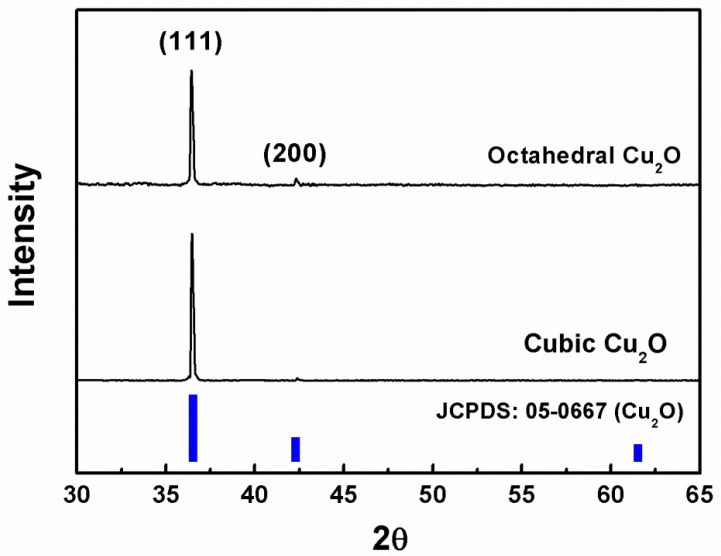
Representative XRD patterns of cubic and octahedral Cu_2_O particles.

The phase of synthesized particles was confirmed by the XRD measurement. The colloids of octahedral Cu_2_O particles were dropped on the slide glass and dried at room temperature. Cubic and octahedral Cu_2_O particles show similar XRD patterns as shown in Figure 2. The relative intensities of {111} and {200} reflections provide information regarding the crystal orientation against the substrate.

**Figure 3.**
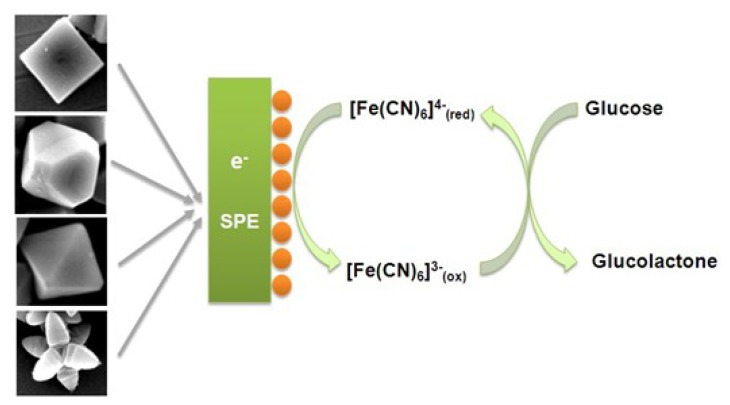
Schematic of Cu_2_O-based electrochemical enzyme-free glucose biosensor.

As an example of application that is suitable for verifying the hypothesis that different surface crystallographic orientations will affect the particles' catalytic performance, the effect of Cu_2_O particles' morphologies on their potential to be used in enzyme-free electrochemical glucose sensor configurations was chosen. Figure 3 shows a schematic of Cu_2_O-based enzyme-free glucose sensors.

**Figure 4.**
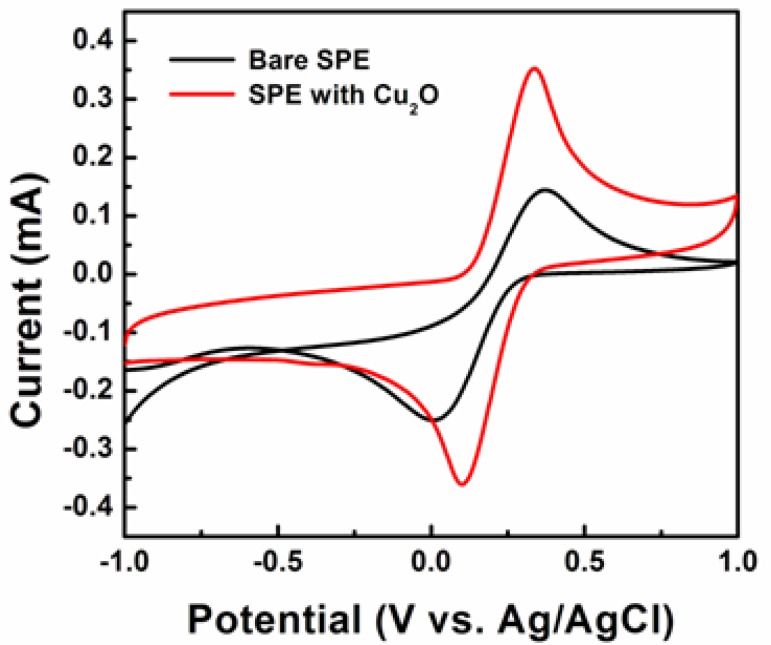
CVs of bare SPE and SPE with hexapod Cu_2_O.

To investigate their morphology-dependent electrochemical properties, electrodes coated with cubic, truncated octahedral, octahedral, and hexapod Cu_2_O particles were prepared. The electrode current responses were measured via a three-electrode system in 0.1 M PBS containing 5 mM [Fe(CN)_6_]^3−/4−^ as an electron mediator. Glucose added to the PBS solution is oxidized to glucolactone by the electron mediator. The amperometric current, generated through this reaction, is indicative of the presence of glucose. Figure 4 shows cyclic voltammograms (CVs) of the bare SPE and the SPE with hexapod Cu_2_O particles in 0.1 M PBS at a scan rate of 100 mV/s.

The redox peaks corresponding to [Fe(CN)_6_]^3−/4−^ couple occur at 330 and 100 mV. The redox peaks of the SPE with Cu_2_O particles show higher intensities than those of the bare electrodes. This shows that Cu_2_O particles hold promise to offer some effectiveness of the enzyme-free sensors based on these materials.

**Figure 5.**
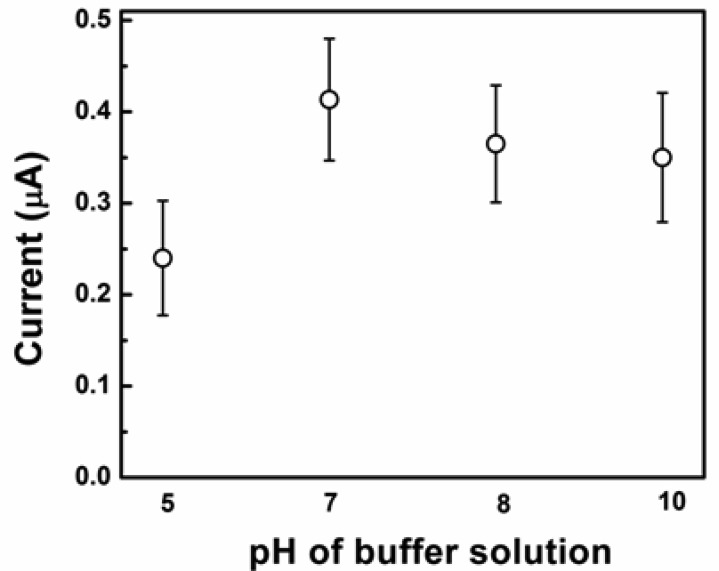
Amperometric responses of the electrode with Cu_2_O to various pH values of PBS (0.1 M) containing 5 mM [Fe(CN)_6_]^3−/4−^ at 50 mV to 5 mM glucose.

The current sensitivity of amperometric sensors is influenced by the applied potential and the pH value of the buffer solution. These variables were optimized to achieve the maximum sensitivity of the sensor. Figure 5 shows the effect of pH values of test PBS solution on the current response at a constant concentration of glucose (5 mM).

**Figure 6.**
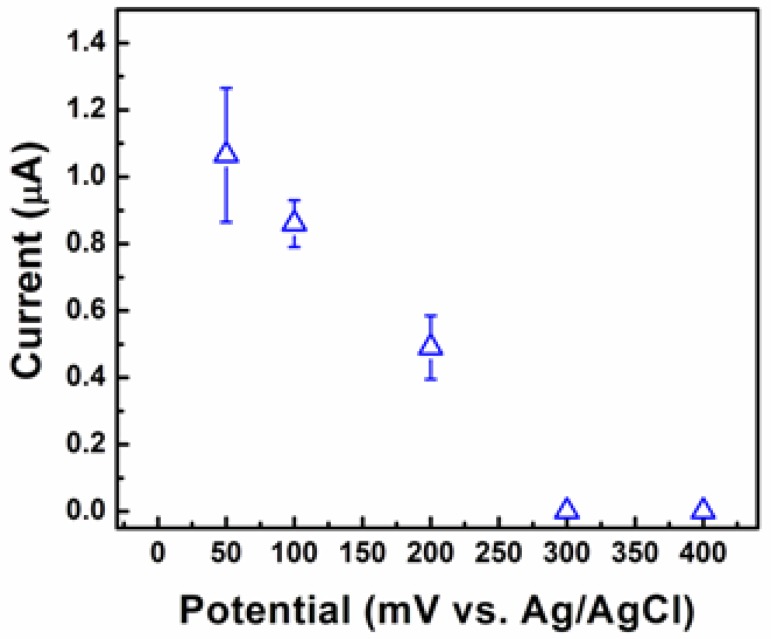
Amperometric responses of the electrode with Cu_2_O to various potential (mV *vs.* Ag/AgCl) of PBS (0.1 M) containing 5 mM [Fe(CN)_6_]^3−/4−^ to 5 mM glucose.

The highest current response was registered at a pH value of 7. To optimize the applied potential three identical electrodes of each particle shape were tested three times at applied potential values ranging from 50 to 400 mV as shown in Figure 6. The maximum value of the current response was registered at 50 mV. These optimized values for pH and applied potential were used for all subsequent testing. To investigate the morphology-dependent electrochemical abilities of the Cu_2_O particles, electrodes coated with Cu_2_O particles of different shapes (cube, truncated octahedron, octahedron, and hexapod) were prepared.

**Figure 7.**
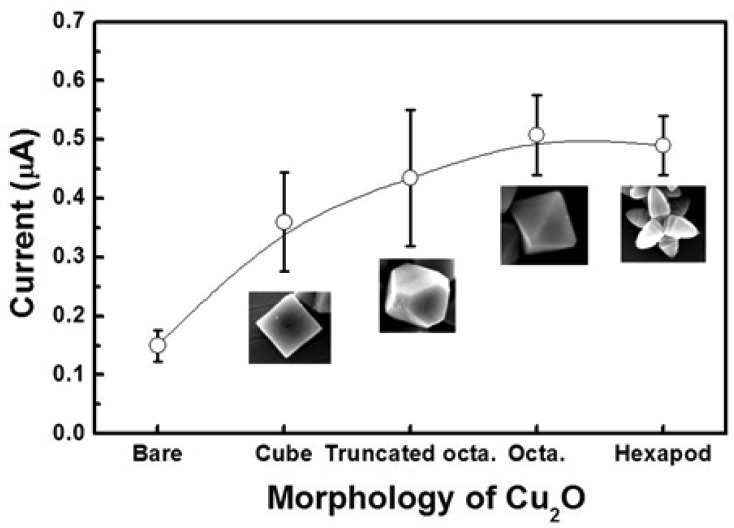
Amperometric responses of electrodes coated with Cu_2_O particles with various morphologies in 0.1 M PBS containing 5 mM [Fe(CN)_6_]^3−/4−^ at an applied potential of 50 mV in the presence of 5 mM glucose.

Figure 7 shows the current responses of these electrodes when glucose was introduced in the system. The current responses were measured three times in 0.1 M PBS containing 5 mM [Fe(CN)_6_]^3−/4−^ at 50 mV and in the presence of 5 mM glucose. The octahedral and hexapod Cu_2_O particles have the {111} crystallographic planes exposed at their surface, while the cubic Cu_2_O particles have the {100} crystallographic planes exposed at their surface.

The results show that the {111} crystallographic orientation is more advantageous in terms of current response than the 100} crystallographic orientation. Interestingly the truncated octahedral Cu_2_O particles, which have both {111} and {100} crystallographic planes exposed at their surface showed a higher current response than the cubic Cu_2_O particles, but somewhat lower than the octahedral and hexapod shaped particles. It can thus be stated that the current responses of the electrodes increase with the area occupied by {111} planes. This finding shows that the {111} crystallographic planes are more effective than the {100} crystallographic planes for catalyzing the electrochemical enzyme free reactions involved in the glucose detection. In these reactions, Cu_2_O particles enhance the electron transfer. It is thus safe to say that the specific electrical conductivity of each shape is affecting the sensitivity of the electrochemical sensing, where electron transfer is critical. From the sensitivity results, we can conclude that the electrical conductivity of the {111} planes is higher than that of the {100} planes in Cu_2_O particles. This result is in good agreement with the report by Kuo *et al.*, in which the authors used *I–V* measurements to show that the {111} facets are more conductive than {100} facets in Cu_2_O particles [30].

**Figure 8.**
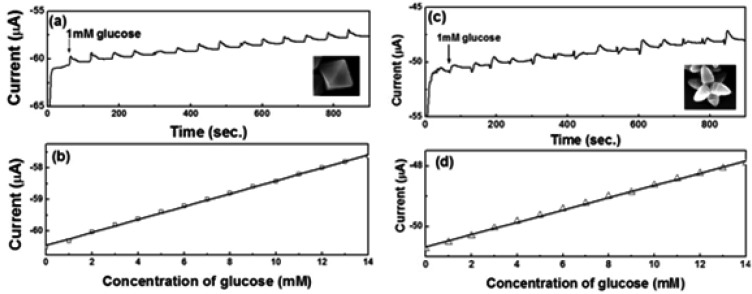
(**a**)/(**c**) Amperometric responses and (**b**)/(**d**) calibration plots (current *vs*. concentration of glucose) with linear regression analysis of octahedral (**a**,**c**) and hexapod (**b**,**d**) Cu_2_O-based electrodes in 0.1 M PBS containing 5 mM [Fe(CN)_6_]^3−/4−^ at an applied potential of 50 mV with successive additions of 1 mM glucose.

Figure 8(a,c) show the amperometric current *vs.* time curves of octahedral and hexapod Cu_2_O-based electrodes, respectively. The current responses were measured by adding 1 mM glucose successively into the test PBS solution containing 5 mM [Fe(CN)_6_]^3−/4−^ at an applied potential of 50 mV. Figure 4(b,d) show the calibration plots with linear regression analysis from Figure 8(a,c), respectively. The current responses of both SPEs showed linear relationships with the concentration of glucose (1–14 mM). The detection limits of SPEs coated with octahedral and hexapod Cu_2_O particles were 0.51 and 0.60 mM of glucose, respectively, determined from the linear graph (signal-to-noise ratio = 3). While the performance of these electrodes are not the best on the market the detection limts are still smaller than the range of physiological blood glucose concentration (2–10 mM). We can conclude that indeed, crystallographic orientation of the facets has an effect on both detection limit and sensitivity, however the sensitivity is affected to a larger extent by crystallographic orientation.

The next question to be answered relates to whether or not doping of metal oxides with noble metals will significantly influence the effect of surface properties of the substrate oxide on the overall doped material electrochemical and catalytic properties. Electrodes modified of Au NPs have been researched as for the non-enzymatic electrochemical detection of glucose [31,32]. We fabricated heterogeneous Au/Cu_2_O particles and evaluated their facet-dependent properties by using similar methods as for bare Cu_2_O particles. Our hypothesis was that the catalytic activity of metal oxides can be enhanced by doping with noble metals, but how does this change with the exposure of various crystallographic planes in Cu_2_O?

**Figure 9.**
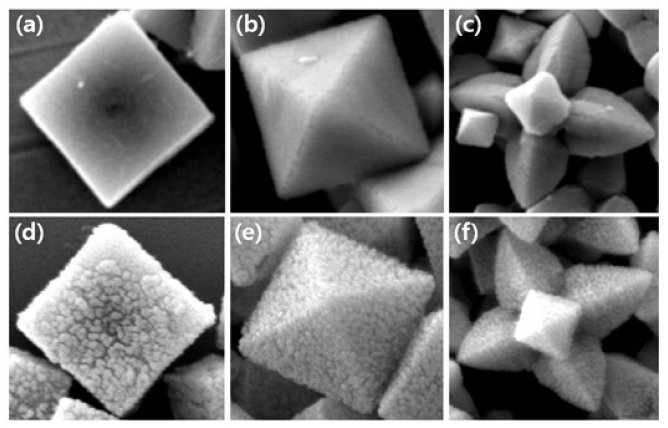
SEM images of (**a**) cubic Cu_2_O; (**b**) octahedral Cu_2_O; (**c**) hexapod Cu_2_O; (**d**) cubic Au/Cu_2_O; (**e**) octahedral Au/Cu_2_O; and (**f**) hexapod Au/Cu_2_O.

We eliminated the need of an electron mediator from the electrochemical sensing configurations, once Au NPs were decorated on the surface of Cu_2_O particles [29]. Au NPs were synthesized on the surface of Cu_2_O particles through the reduction of AuCl_4_^−^ anions by Cu_2_O, according to Equation (1). Figure 9 shows SEM images of cubic (a,d), octahedral (b,e), and hexapod (c,f) Cu_2_O particles before and after Au decorating.

**Figure 10.**
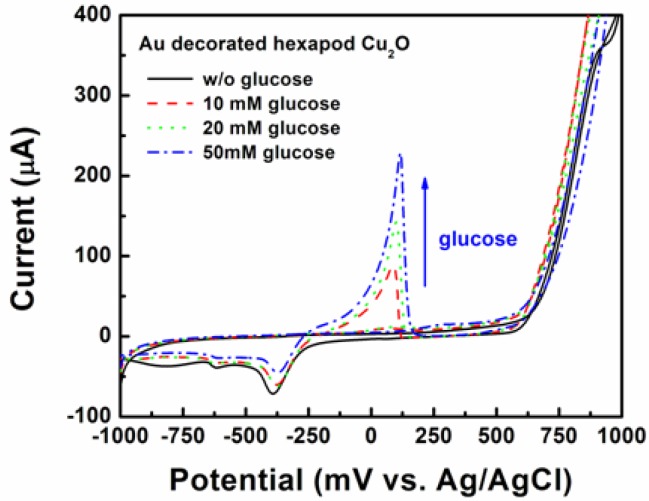
Cyclic voltammograms of hexapod Au/Cu_2_O-based electrode in 0.5 M NaOH with additions of 0, 10, 20, and 50 mM glucose at a scan rate of 10 mV/s.

To test the hypothesis that Au doping of Cu_2_O will enhance their electrocatalytic properties and capacity to be incorporated in enzyme-free electrochemical sensing configuration, Au/Cu_2_O particles were deposited on screen printed electrodes and tested for the detection of glucose. Since Cu_2_O alone did not seem to offer the best detection limit for glucose, the next question to be answered was whether or not synergistically combining the good electrocatalytic ability towards glucose oxidation of Au NPs, with the good electrical conductivity of Cu_2_O particles with {111} faces will lead to significant improvements in this area. The answer to this question will be significant from the point of view of understanding how doping with noble metals might influence oxides’ performance and whether or not the initial shape effects are still maintained. Figure 10 shows the CVs of the SPE with Au decorated hexapod Cu_2_O in 0.5 M NaOH solution in the absence and presence of glucose (10, 20, and 50 mM) at a scan rate of 10 mV/s.

It is obvious that the SPE shows no peak corresponding to glucose oxidation (the black solid line in Figure 6). In the presence of glucose, the electrode shows the indicative peak of glucose oxidation at 100 mV. As the glucose concentration increased from 10 to 50 mM, the peak intensity also increased. On the other hand, the peak currents corresponding to AuOH reduction at −350 mV decreased, due to the fact that certain amounts of AuOH were used for glucose oxidation as the glucose concentration increased [32].

**Figure 11.**
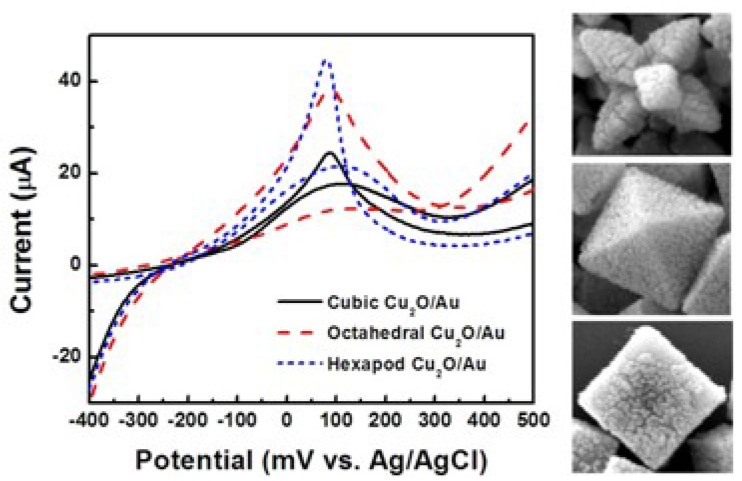
Cyclic voltammograms of electrodes coated with Au decorated cubic, octahedral, and hexapod Cu_2_O in 0.5 M NaOH solution in the presence of 10 mM glucose and corresponding SEM images of the particles.

In the next step, the influence of the crystallographic orientation of Cu_2_O particles in the Au/Cu_2_O configuration, on the effectiveness of glucose oxidation was investigated. Figure 11 shows CVs of SPEs coated with Au decorated cubic, octahedral, and hexapod Cu_2_O, which were tested in 0.5 M NaOH solution, and in the presence of 10 mM glucose. The octahedral and hexapod Au/Cu_2_O particles showed higher peak currents than the cubic Au/Cu_2_O particles. This is following the same trends described above for bare Cu_2_O particles, in terms of the influence of the conductivities of {111} and {100} facets.

**Figure 12.**
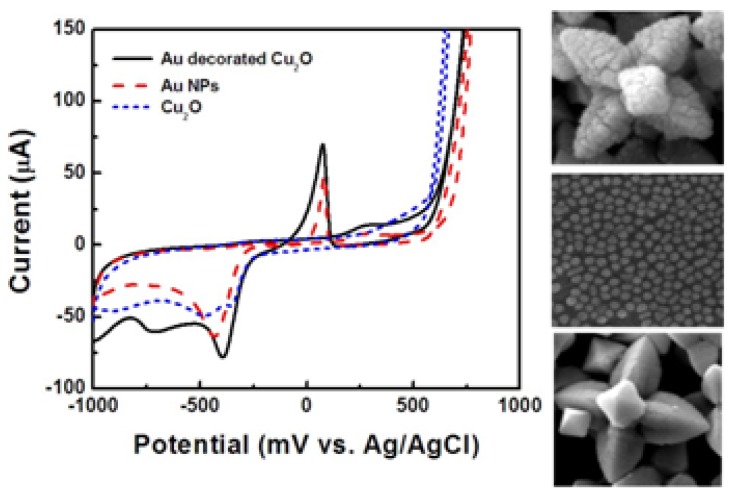
Cyclic voltammograms of electrodes coated with hexapod Au/Cu_2_O particles, Au nanoparticles, and hexapod Cu_2_O in 0.5 M NaOH solution, and in the presence of 10 mM glucose and corresponding SEM images of the particles.

To identify the peak corresponding to the glucose oxidation, cyclic voltammograms coated with Au decorated hexapod Cu_2_O particles, Au nanoparticles, and uncoated hexapod Cu_2_O were tested. The testing solution contained 0.5 M NaOH and 10 mM glucose as shown in Figure 12. While the electrodes with hexapod Cu_2_O particles showed no peak for glucose oxidation given the absence of an electron mediator, the electrodes with Au NPs and Au/Cu_2_O particles showed peaks corresponding to glucose oxidation at 100 mV. The peak current corresponding to Au/Cu_2_O was higher than that corresponding to only Au nanoparticles. This indicates that the best electrochemical performance is offered by doping Cu_2_O particles with Au rather than using Au nanoparticles alone. As mentioned above, the current response of amperometric devices is affected by the applied potential. To find the optimum potential, the current responses of electrodes coated with Au/Cu_2_O particles were obtained at applied potentials ranging from −700 to 700 mV.

**Figure 13.**
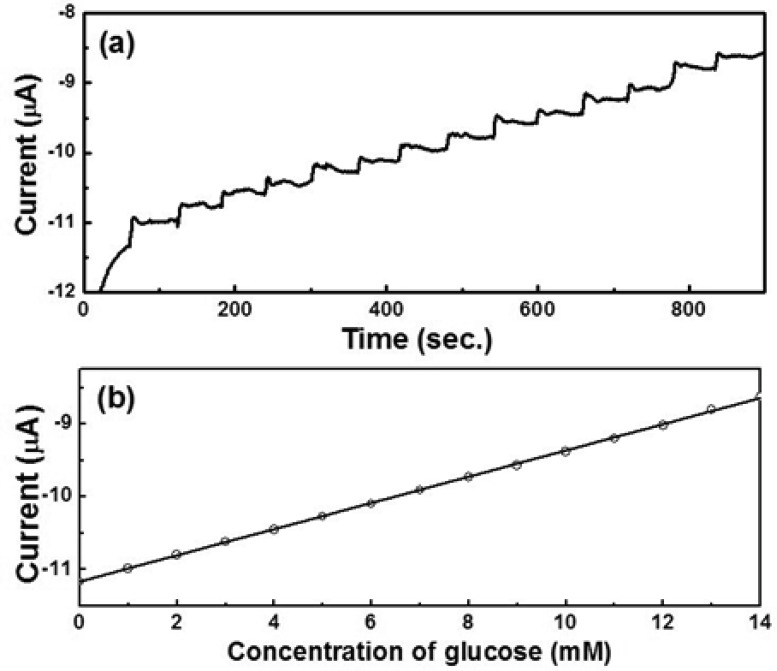
(**a**) Amperometric responses and (**b**) calibration plot (current *vs.* concentration of glucose) with linear regression analysis of hexapod Au/Cu_2_O-based electrodes in 0.5 M NaOH at an applied potential of −600 mV and with successive additions of 1 mM glucose.

The current response of the SPEs showed the highest value at −600 mV (data not shown). Thus, Au/Cu_2_O-based enzyme-free glucose sensors were tested at −600 mV. Figure 13(a,b) show the amperometric responses *vs*. time curve and the calibration plot, respectively, of the electrode coated with hexapod Au/Cu_2_O particles in 0.5 M NaOH, at an applied potential of −600 mV, with successive additions of 1 mM glucose.

The current responses are linear with the glucose concentration in a wide range from 1 to 14 mM (R^2^ = 0.999). The detection limit of the sensor based on Au/Cu_2_O particles were 0.21 mM glucose, which is lower than that for the undoped hexapod Cu_2_O particles (detection limit = 0.6 mM), determined from the linear graph (signal-to-noise ratio = 3). We can conclude that the experimental results converge to the idea that the exposure of {111} planes in Cu_2_O particles, together with Au doping is the most effective configuration from the point of view of the electrocatalytical properties. This is due to synergistic effects brought in by the increased conductivity of Cu_2_O {111} crystallographic planes, coupled with the enhanced electron transfer capabilities of Au NPs. While our results established that the shape and facet crystallographic orientation indeed plays a role on the materials ‘electrocatalytic activity in both doped and un-doped oxides, the inherent redox properties still maintain the dominant influence. Having the capacity to control the shapes and surface orientation of various oxide particles can lead to opportunities in fine tuning certain properties, especially those that are affected by the electrical conductivity.

## 4. Conclusions

Cu_2_O and Au/Cu_2_O particles of various shapes (cube, truncated octahedron, octahedron, and hexapod) were synthesized and their morphology-dependent electrochemical properties were tested in an example configuration set up for the detection of glucose. The octahedral and hexapod Cu_2_O that have a {111} crystallographic orientation exposed at their surface, showed improved electrocatalytical properties than the cubic Cu_2_O, which have {100} orientations exposed at their surface. This suggests that the {111} orientations are more electrical conductive than the {100} facets in Cu_2_O particles. While we did not attempt to obtain the best glucose sensing on the market, but to merely use glucose sensing as a well studied model, the electrodes fabricated with octahedral and hexapod Cu_2_O particles showed detection limits of physiological relevance concentrations. While the detection limit is slightly affected particle orientation, there is a larger effect on the sensitivity. Heterogeneous systems where Au NPs were deposited on the surface of Cu_2_O particles were also tested. The hypothesis that surface orientation influences observed in the electrochemical configuration with solely Cu_2_O particles maintained in Au/Cu_2_O systems was tested. These materials showed that the hexapod shape of Au/Cu_2_O synergetically combined the effect of the electrocatalytic ability towards glucose oxidation of Au NPs, with the increased electrical conductivity of {111} facets of the hexapod Cu_2_O particles. While the inherent physical and chemical properties of the materials themselves maintain the dominant role in their electrocatalytic capacity, having the opportunity to control the shapes and surface orientation of various particles can assist in fine tuning certain properties, especially those where the electrical conductivity is critical.

## Figures and Tables

**Table 1.** Raw materials for the synthesis of Cu_2_O particles.

	**Cubes**	**Truncated octahedra**	**Octahedra**	**Hexapods**
**DI water**	9.5 mL	9.2 mL	9.0 mL	9.05 mL
**0.1 M CuCl_2_**	0.1 mL	0.1 mL	0.1 mL	0.1 mL
**1.0 M NaOH**	0.3 mL	0.3 mL	0.3 mL	0.2 mL
** SDS**	0.087 g	0.087 g	0.087 g	0.087 g
**0.2 M NH_2_OH·HCl**	0.1 mL	0.4 mL	0.6 mL	0.65 mL
